# MicroRNA-143 Targets MACC1 to Inhibit Cell Invasion and Migration in Colorectal cancer

**DOI:** 10.1186/1476-4598-11-23

**Published:** 2012-04-25

**Authors:** Yu Zhang, Zhongqiu Wang, Min Chen, Liang Peng, Xinying Wang, Qunying Ma, Fengli Ma, Bo Jiang

**Affiliations:** 1 Guangdong Provincial key laboratory of Gastroenterology, Department of Gastroenterology, Nangfang Hospital, Southern Medical University, 1838 North Guangzhou Road, Guangzhou, Guangdong Province 510515, P.R. China

**Keywords:** miR-143, MACC1, Colorectal cancer, Invasion, Migration

## Abstract

**Background:**

MicroRNAs (miRNAs) have been suggested to play a vital role in tumor initiation and progression by negatively regulating oncogenes and tumor suppressors. Quite recently, studies have identified some miRNAs operating to promote or suppress tumor invasion or metastasis via regulating metastasis-related genes, providing potential therapeutic targets on anti-metastasis strategy. Metastasis-associated in colon cancer-1 (MACC1) has been newly identified to express highly in colorectal cancer (CRC) and promote tumor metastasis through transactivating metastasis-inducing HGF/MET signaling pathway. In this study, we investigated whether miRNA 143 is involved in the regulation of MACC1 and thus plays a functional role in CRC.

**Results:**

Using both in silico prediction and western blot assay, we found the previously reported tumor suppressive miR-143 targeted MACC1 in CRC. The direct interaction between them was confirmed by 3' UTR luciferase reporter gene. In concordance with the inhibitory effects induced by siRNA mediated knockdown of MACC1, restoration of miR-143 by mimics in SW620 cells significantly attenuated cell growth, migration and invasion. It is notable that combined treatment of miR-143 mimics and MACC1 siRNA induced synergistic inhibitory effects compared to either miR-143 mimics or MACC1 siRNA treatment alone. Conversely, reduction of miR-143 by inhibitors in SW480 cells apparently stimulated these phenotypes. Furthermore, we observed that miR-143 level was inversely correlated with MACC1 mRNA expression in CRC tissues.

**Conclusions:**

Our findings newly described miR-143/MACC1 link and provided a potential mechanism for MACC1 dysregulation and contribution to CRC cell invasion. It may help to estimate the therapeutic utility of miR-143 in CRC.

## Introduction

Colorectal cancer (CRC) is the third most common cancer and the second leading cause of cancer-related deaths in western countries [1], with very poor prognosis and high possibilities of tumor invasion and migration. Although invasion and migration have been acknowledged as the most lethal attributes of solid tumors, the molecular mechanism underlying them is still limited.

Recently, growing evidences have supported the cancer-related effects of miRNAs, a newly discovered class of non-coding small RNA which functions through negatively regulating a variety of gene expression. Mature miRNAs exert effects by integrating into an RNA-inducing silencing complex (RISC) and binding to specific complementary sites within 3' untranslated regions (3'UTR) of their target genes mRNA, to inhibit translation or directly induce degradation [2-5]. Bioinformatic algorithms assess that all the human miRNAs may regulate up to 30% of human genes which represent the majority of genetic pathways [6,7]. Many studies have identified specific miRNAs expression profiles of multiple cancer types compared to those of normal adjacent tissues. Depending on cellular contexts and target genes that they regulate, miRNAs may function as tumor suppressors or oncogenes [8,9]. Among these functional miRNAs, miR-143 has been demonstrated to significantly decrease in multiple cancer types and play a role of tumor suppressor. For instance, loss of miR-143 was observed in bladder cancer, whereas enhanced expression of miR-143 induced growth suppression in bladder cancer cells through downregulation of Erk5 and Akt expression at translational level [10]. Moreover, miR-143 was validated to inhibit prostate cancer cells proliferation and migration and enhance their sensitivity to docetaxel through suppressing K-RAS [11]. In CRC, Micheal et al. [12] reported that miR-143 consistently displays reduced steady-state levels at the adenomatous and cancer stages of CRC. K-RAS and DNMT3A gene were subsequently identified to be regulated by miR-143, which partly explained the inhibitory effect of miR-143 on CRC growth [13,14]. Besides, a clinical study found that, compared to adjacent normal colon tissues, miR-143 exhibited a lower expression in colorectal liver metastases as well as that in primary tumor [15], which implied the involvement of miR-143 in CRC invasion and metastasis.

Metastasis-associated in colon cancer-1 (MACC1), a newly identified CRC tumorigenesis and metastasis related gene, has recently been identified to act as a key activator of the metastasis-inducing HGF/Met signaling pathway, promoting proliferation, invasion and HGF-induced scattering of CRC cells in cell culture and tumor growth and metastasis in xenograft models [16]. Several studies demonstrated that, besides in CRC, MACC1 could serve as an independent prognostic marker of tumor invasiveness and metastasis in some other cancer types [17-19]. Existing data regarding the involvement of MACC1 in tumor metastasis also suggest its particular therapeutic impact.

In the present study, we confirmed the regulatory relationship between miR-143, a known tumor suppressive miRNA, and a new oncogene, MACC1. We provided evidences that miR-143 could impede CRC cell invasion and migration, at least partly by targeting MACC1. Furthermore, the correlation between miR-143 and MACC1 expression level in CRC was determined.

## Results

### miR-143 is frequently downregulated in CRC tissues and cell lines

**Figure 1:**
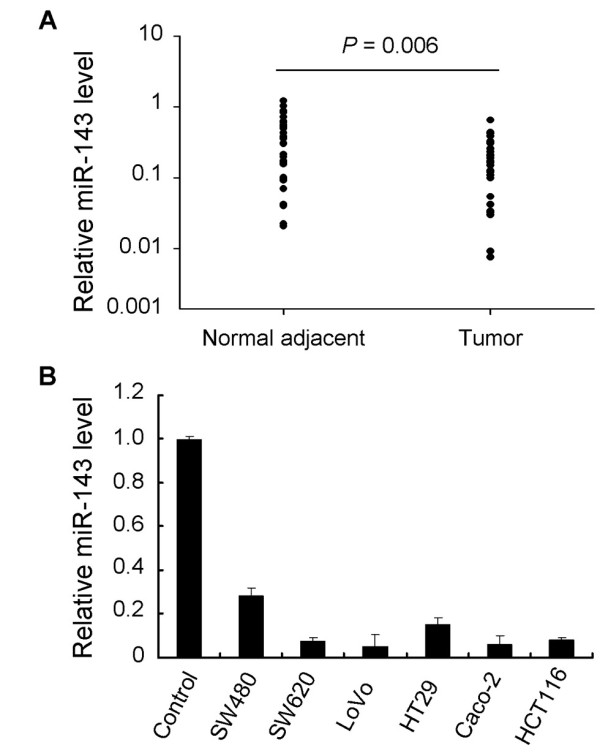
**Decreased expression of miR-143 in both primary CRC tissues and CRC cell lines**. (A) Decreased miR-143 expression (log10 scale at Y axis) in CRC tissues compared to the adjacent normal colonic tissues from a panel of 30 CRC patients (Wilcoxon's paired test, P = 0.006). (B) Significant loss of miR-143 expression in six CRC cell lines in comparison with control normal colonic mucosa pooled from 3 healthy individuals. Figure is representative of 3 experiments with similar results.

We performed SYBR green quantitative PCR analysis to detect the expression level of miR-143 in CRC tissues and cell lines. In the large panel of 30 cases of primary CRC tissues and their adjacent normal colonic tissues, our results showed that miR-143 was significantly decreased in 23 (76.6%) CRC tissues when compared with that in the paired adjacent normal tissues (Figure 1A). In addition, we extended our test to six human CRC cell lines. The total six cell lines showed a notable loss of miR-143, whereas the control normal colonic mucosa pooled from 3 healthy individuals expressed a strong level of it (Figure 1B).

### miR-143 directly targets MACC1

**Figure 2:**
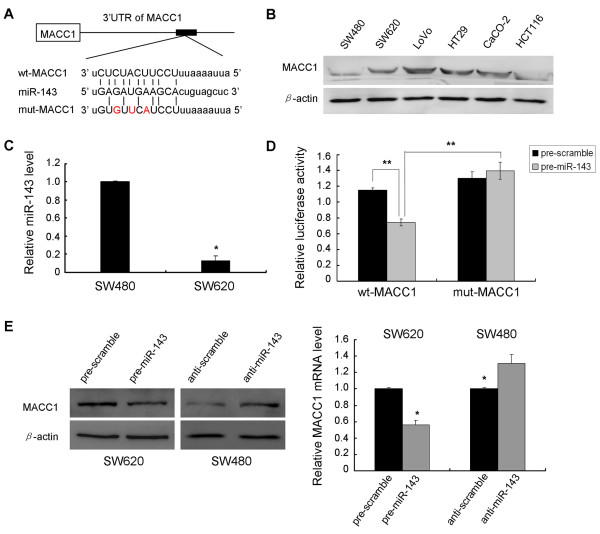
**miR-143 directly targets MACC1 by binding to its 3'UTR**. (A) The predicted miR-143 binding site within MACC1 3'UTR and its mutated version by site mutagenesis are as shown. (B) Variable MACC1 expression in six CRC cell lines was obtained by Western blot. β-actin was used as the loading control. (C) Non-metastatic SW480 cells expressed significantly higher level of miR-143 compared with its metastatic counterpart SW620 cells (* P < 0.05). (D) The repression of luciferase activity by MACC1 3'UTR was dependent on miR-143. Mutated MACC1 3'UTR abrogated miR-143 mediated repression luciferase activity (** P < 0.01). (E) In comparison with scramble controls, elevated expression of miR-143 by mimics inhibited MACC1 expression at both mRNA and protein level, while reduction of miR-143 by inhibitors moderately restored MACC1 expression (* P < 0.05). Figure is representative of 3 experiments with similar results.

Using online miRNA target prediction databases (miRNA.org and Targetscan), we hypothesized that metastasis-associated in colon cancer-1 (MACC1), which has been newly identified as a positive prognostic indicator of metastasis formation in CRC [16], was a target of miR-143 (Figure 2A). We first examined the MACC1 expression profile in the panel of six CRC cell lines. Western blot analysis showed that both the metastasis-derived LoVo and SW620 cells had notable MACC1 expression as well as HT29 and Caco-2 cells (Figure 2B). SW480 and SW620 cell lines are derived from the primary colon adenocarcinoma and a node metastasis resected from a single patient respectively [20]. It has been reported that SW620 cells were more highly metastatic than some other CRC cell lines including SW480 cells [21,22]. Since SW480 cells showed significantly lower MACC1 protein expression (Figure 2B) and higher miR-143 expression (Figure 2C) compared to SW620 cells, we selected these two cell lines to verify our hypothesis. We transfected SW620 cells or SW480 cells with miR-143 mimics or inhibitors at a concentration of 50 nM. Western blot showed that, at 48 h after transfection, the enhanced miR-143 in SW620 cells significantly repressed MACC1 protein expression compared to cells transfected with scramble control (Figure 2E). Relatively, downregulation of miR-143 by inhibitors in SW480 cells led to a moderate increase of MACC1 protein level (Figure 2E). Meanwhile, apparent alterations of MACC1 mRNA expression were also observed by quantitative PCR (Figure 2E). It suggested a potential regulation of MACC1 by miR-143. To further investigate if the predicted binding site of miR-143 to 3'UTR of MACC1 is responsible for this regulation, we cloned the 3'UTR of MACC1 downstream to a luciferase reporter gene (wt-MACC1), its mutant version (mut-MACC1) by the binding site mutagenesis was also constructed. We co-transfected wt-MACC1 vector and miR-143 mimics or scramble control into HEK293 cells. The luciferase activity of miR-143 transfected cells was significantly reduced compared to scramble control cells (Figure 2D). Moreover, miR-143-mediated repression of luciferase activity was abolished by the mutant putative binding site (Figure 2D). These results suggested miR-143 could inhibit MACC1 expression at transcriptional level.

### Effect of miR-143 on CRC cell growth, migration and invasion

**Figure 3:**
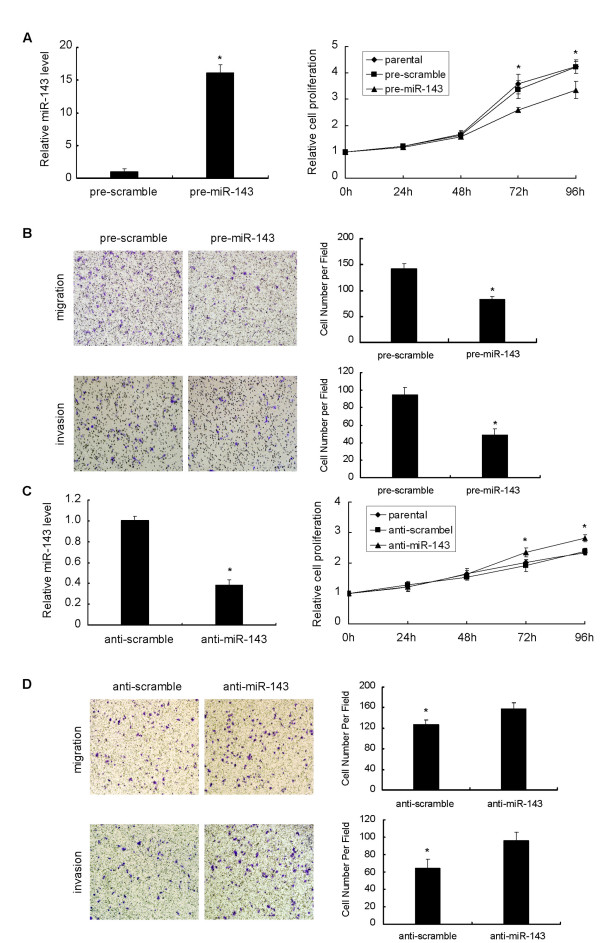
**Effects of miR-143 on proliferation, migration and invasion of SW620 and SW480 cell lines**. (A) Ectopic expression of miR-143 by transfecting miR-143 mimics significantly reduced proliferation of SW620 cells, in comparison with parental and scramble controls (* P < 0.05). (B) Ectopic expression of miR-143 notably inhibited cell migration and invasion of SW620 cells (100 × magnification, * P < 0.05). Inversely, inhibition of miR-143 expression by transfecting miR-143 inhibitors simultaneously (C) promoted proliferation (*P < 0.05) and (D) stimulated cell migration and invasion of SW480 cells, compared with parental and scramble controls (100 × magnification, * P < 0.05). Figure is representative of 3 experiments with similar results.

To validate if miR-143 regulates CRC cell growth, we performed a proliferation assay by transfecting miR-143 mimics or scramble control into SW620 cells. It showed that the increased expression of miR-143 induced significant inhibition on cell growth (Figure 3A). Correspondingly, after transfected with miR-143 inhibitors, SW480 cells presented stimulated cell growth compared to scramble control (Figure 3C). Cell motility of transfected cells was also evaluated by migration and invasion assays. As shown in Figure 3B, compared to the scramble control, miR-143 mimics transfected SW620 cells exhibited significant impairment of migratory ability. The corresponding effect on invasive ability was also observed in parallel invasion assay. Inversely, downregulation of miR-143 in inhibitors transfected SW480 cells apparently promoted cell migration and invasion ability (Figure 3D).

### Knockdown of MACC1 expression repress CRC cell growth, migration and invasion

**Figure 4:**
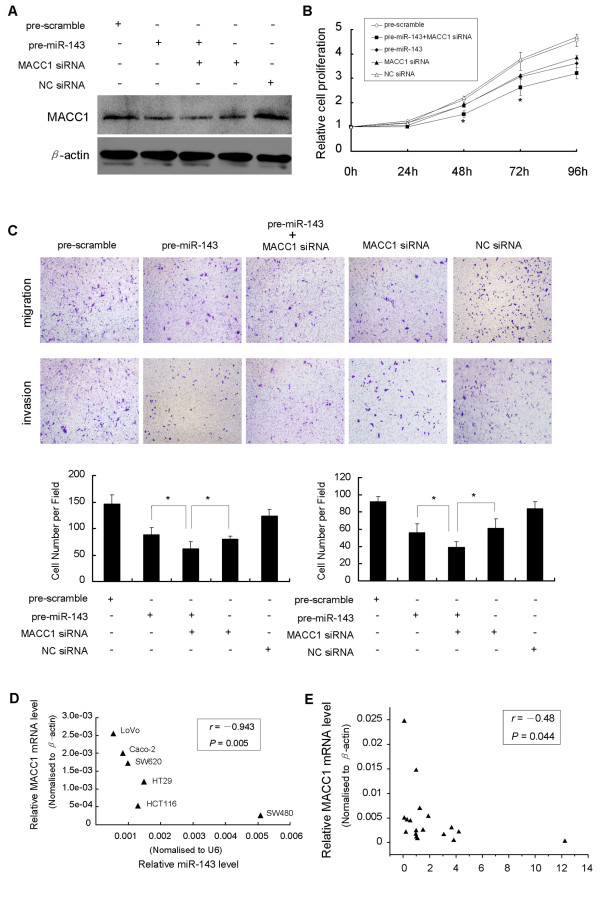
**Functional effects of MACC1 downregulation on SW620 cells**. Inverse correlation between miR-143 and MACC1 expression in CRC. (A) Effectively suppression of MACC1 protein expression by MACC1 siRNA and miR-143 mimics respectively and combinedly. Note the more efficient suppression of MACC1 induced by combined treatment. Suppression of MACC1 simultaneously resulted in (B) significant inhibition of cell growth and (C) cell migration and invasion (100 × magnification) of SW620 cells compared with negative controls. Note the synergistic inhibitory effect induced by combination of MACC1 siRNA and miR-143 mimics, compared with either of them alone (*P < 0.05). The correlation between miR-143 and MACC1 mRNA expression by scatter plot. (D) An inverse correlation was observed in six CRC cell lines (Spearman' s correlation, r = -0.943, P = 0.005), and (E) also observed in another independent panel of 9 paired CRC tissues and adjacent normal tissues (Spearman's correlation, r = -0.48, P = 0.044). Figure is representative of 3 experiments with similar results.

To further confirm the potential relationship between miR-143 and the downstream gene MACC1, we tested cell growth and motility under the condition of siRNA mediated knockdown of MACC1 gene. Once MACC1 expression was effectively depressed by siRNA (Figure 4A), transfected SW620 cells exhibited decreased cell growth (Figure 4B), impaired cell migration and invasion ability (Figure 4C), which was in consistent with the inhibitory effects induced by downregulation of miR-143. Furthermore, when treating SW620 cells with MACC1 siRNA in combination with miR-143 mimics, we observed synergistic inhibitory effects on MACC1 expression (Figure 4A), cell growth (Figure 4B), cell migration and invasion ability (Figure 4C), compared to either MACC1 siRNA or miR-143 mimics treatment alone. Taken together, these results indicated that miR-143 functions as a potent tumor suppressor through regulating MACC1 expression.

### Correlation of expression between miR-143 and MACC1

To confirm the relevance between miR-143 and MACC1 expression, we investigated the expressions of miR-143 and MACC1 mRNA in the six CRC cell lines and the small panel of 9 paired primary CRC and normal adjacent colonic tissues. In all CRC cell lines, an inverse correlation of expression between miR-143 and MACC1 mRNA was observed (Figure 4D). In the analyzed panel of 9 CRC patients, all the tumor tissues showed a notable decreased expression of miR-143 compared to their matched adjacent normal tissues. We also observed an inverse correlation between miR-143 and MACC1 expression in CRC tissues and their adjacent normal tissues (Figure 4E).

## Discussion

Most of deaths from cancer are caused by complications arising from metastasis. Therefore, targeting metastatic disease is a pivotal anti-cancer strategy. Studies on tumor invasion and metastasis have revealed the critical role of miRNA in these processes by the mechanism that miRNA could regulate a variety of genes pivotal for invasion or metastasis [23,24]. Quite recently, some miRNAs have been identified to promote [25-27] or suppress [28-30] tumor invasion or metastasis, providing potential therapeutic targets on anti-metastasis strategy. In CRC, miR-21 has been demonstrated to enhance cellular invasion, intravasation and metastasis through post-transcriptionally suppressing PDCD4 gene [31]. By targeting Cdc42, miR-137 exerted inhibitory effect on CRC cell invasion [32]. Of these deregulated miRNA in CRC specific miRNA profile, miR-143 was steadily downregulated in the whole CRC succession from adenomatous to cancer [12]. Further studies showed that miR-143 was tumor invasion front-specific downregulated in CRC, and associated with aggressive mucinous phenotype in CRC [33,34]. In a mouse colorectal carcinoma xenograft study, overexpression of miR-143 could impair tumor growth by induction of apoptosis and inhibition of proliferation [35]. Based on these findings, we speculated miR-143 might be involved in CRC metastatic processes. Finally, we confirmed the link between miR-143 and MACC1, a positive CRC metastasis related gene, and found that miR-143 inhibited CRC cell invasion and migration via targeting MACC1.

Our results obtained from quantitative PCR validation that miR-143 was commonly downregulated in CRC cell lines and in 32 out of 39 (82.1%) enrolled CRC patients, were in consistent to previous studies. Of these analyzed CRC tissues, we found that miR-143 downregulation was not associated with clinical characteristics of patients (data not shown). Since SW620 cells had a pretty lower expression of miR-143 compared to SW480 cells, which were derived from the same patient as SW620, we selected these two cell lines to perform further in vitro function assays. We restored miR-143 expression in SW620 cells and found that miR-143 inhibited cell proliferation, invasion and migration. On the contrary, when transfected with miR-143 inhibitors, SW480 cells exhibited stimulated proliferation as well as invasion and migration capabilities. These findings suggested that miR-143 was involved in the processes of metastasis.

MACC1, reported by Stein et al. [16], was identified to be an independent prognostic indicator of metastasis formation and metastasis-free survival. MACC1-based positive and negative prediction of metachronous metastasis was correct to 74% and 80% respectively [36]. Mechanically, MACC1 transcriptionally activated Met to promote HGF/Met signaling pathway, which has been reported essential for tumorigenesis and metastasis. Our results confirmed a vital molecular relationship between miR-143 and MACC1. We showed that, at both mRNA and protein level, upregulation of miR-143 expression in SW620 cells effectively suppressed MACC1 expression, whereas, in SW480 cells, downregulation of miR-143 moderately promoted MACC1 expression. It suggested a potential inverse relevance of miR-143 and MACC1 in CRC. Loss-of-function study of MACC1 by siRNA-mediated knockdown demonstrated suppressive effects on cellular proliferation, invasion and migration, which were also verified in gain-of-function study by enhanced miR-143 expression. Furthermore, by luciferase reporter study, we verified that miR-143 directly target MACC1 gene through binding to specific complementary site within its 3' untranslated region. In addition, the inverse correlation of expression between miR-143 and MACC1 expression in CRC cell lines and tissues indicated that, decreased expression of miR-143 may account for upregulation of MACC1 in CRC development. Taken together, these findings sufficiently consolidated that miR-143 played a suppressive role in cellular proliferation, migration and invasion, at least, in part due to directly inhibiting MACC1 expression.

Activation of HGF/Met signaling pathway leads to multiple malignant processes, including cell growth, EMT, angiogenesis, cell motility, invasiveness, and metastasis [37]. Stein et al. [16] reported a MACC1-driven positive feedback loop in CRC metastasis that MACC1 translocated into nuclear following HGF treatment and bound to the Met promoter to activate HGF/Met signaling. In xenograft model study, they showed that knockdown of MACC1 by si/shRNA resulted in significant reduction of tumor size and number of liver metastases. That suggested targeting MACC1 was a promising strategy in prevention of CRC metastasis. Our study revealed the inhibitory effect of miR-143 on MACC1, and partly elucidated a potential molecular mechanism by which miR-143 participated in CRC agrressiveness.

More recently, Hurst et al. [38] proposed a novel field of cancer-related miRNAs termed metastamir that are associated with metastatic processes. For example, miR-21 is a mastermind of metastasis that promotes cell survival, migration, invasion, in vivo intravasasion and metastasis [39-41], whereas miR-200 family is delinquent whose absence contributes to EMT phenotype [42-44]. These metastamir represent potential candidate cancer prognostic markers and therapeutic targets for metastatic cancers. Our findings suggest that miR-143 could function as a metastamir via targeting MACC1.

In conclusion, we newly described miR-143/MACC1 link and provided a potential mechanism for MACC1 dysregulation and contribution to CRC cell invasion. As a result, restoration of miR-143 expression could have an important implication for the clinical management of CRC.

## Materials and methods

### Tissue samples, cell lines and cell transfection

A total of 39 pairs of primary CRC and their matched adjacent normal colonic epithelial tissues were collected and randomly divided into a large panel of 30 pairs and a small panel of 9 pairs. All samples were obtained from patients who underwent surgical resections at Nanfang Hospital (Guangzhou, China) and snap-frozen in liquid nitrogen, then stored at -80°C for further use. This project was approved by the Ethic Committee of Nanfang hospital.

Six human colorectal cancer (CRC) cell lines, including LoVo, HT29, SW480, SW620, Caco-2 and HCT116, were purchased from American Type Culture Collection. Cells were grown routinely in RPMI-1640 medium (Invitrogen, CA, USA) supplemented with 10% fetal bovine serum (Gibco, CA, USA) and cultured in a 37°C humidified atmosphere of 5% CO2.

Ectopic expression of miR-143 in cells was achieved by transfection with miR-143 mimics or inhibitors (Genepharma, Shanghai, China) using Lipofectamine2000 (Invitrogen, CA, USA). Knockdown of MACC1 was performed using MACC1 siRNA (Genepharma, Shanghai, China). Cells were plated in 6-well clusters or 96-well plates and transfected for 24 h or 48 h. Transfected cells were used in further assays or RNA/protein extraction.

### RNA extraction and SYBR green quantitative PCR analysis

Total RNA was extracted from cells using Trizol reagent (Invitrogen, CA, USA). Mature miR-143 expressions in cells were detected using a Hairpin-it TM miRNAs qPCR kit (Genepharma, Shanghai, China). Expression of RNU6B was used as an endogenous control. MACC1 expression was measured by SYBR green qPCR assay (Takara, Dalian, China). Data were processed using 2-ΔΔCT method.

### CCK-8 cell proliferation assay

Cell proliferation rates were measured using Cell Counting Kit-8 (CCK-8) (Beyotime, Hangzhou, China). 0.5 × 104 cells were seeded in each 96-well plate for 24 h, transfected with the indicated miRNA or siRNA, and further incubated for 24 h, 48 h, 72 h and 96 h respectively. 10 μl CCK-8 reagent was added to each well at 1 h before the endpoint of incubation. OD450nm value in each well was determined by a microplate reader.

### Cell invasion and migration assay

The invasive and migratory potential of cells was evaluated using transwell inserts with 8 μm pores (Coring, NY, USA). For invasion assay, at 24 h after transfection, 3.0 × 105 cells in serum free medium were added to each upper insert pre-coated with matrigel matrix (BD, NJ, USA). 500 μl 10% FBS medium was added to the matched lower chamber. After 48 h incubation, non-invaded cells were removed from the upper surface of the transwell membrane with a cotton swab, and invaded cells on the lower membrane surface were fixed in methanol, stained with 0.1% crystal violet, photographed, and counted. For migration assay, the procedures were similar, except that 2 × 105 cells were added into the insterts without matrix gel pre-coated. Six random fields at 100 × magnification for each insert were counted. Inserts were conducted in triplicate in three separate experiments.

### Western blot analysis

Immunoblotting was performed to detect the expression of MACC1 in CRC cell lines. Cultured or transfected cells were lysed in RIPA buffer with 1% PMSF. Protein was loaded onto a SDS-PAGE minigel and transferred onto PVDF membrane. After probed with 1:1000 diluted rabbit polyclonal MACC1 antibody (Abcam, MA, USA) at 4°C overnight, the blots were subsequently incubated with HRP-conjugated secondary antibody (1:5000). Signals were visualized using ECL Substrates (Millipore, MA, USA). β-actin was used as an endogenous protein for normalization.

### Luciferase reporter assay

A fragment of 3'UTR of MACC1 (1022 bp) containing the putative miR-143 binding site was amplified by PCR using following primers:

wt-MACC1 (forward) 5' CCG***CTCGAG***CACCAGTAAAACAAGGAACTTG 3'

wt-MACC1 (reverse) 5' GAAT***GCGGCCGC***TTTACAGAAACAAATGCAATGTTAC 3'

The PCR product was subcloned into a psiCHECK-2 vector (Promega, Madison, WI) immediately downstream to the luciferase gene sequence. A psiCHECK-2 construct containing 3'UTR of MACC1 with a mutant seed sequence of miR-143 was also synthesized using the primers:

mut-MACC1 (forward) 5' TTAAAATTTCCTACTTGTGTATAGAAATGGAAAG 3'

mut-MACC1 (reverse) 5' ACAAGTAGGAAATTTTAATACAGTGGTTCTC 3'

All constructs were verified by DNA sequencing. HEK293 cells were plated in 96-well clusters, then co-transfected with 100 ng constructs with or without miR-143 precursors. At 48 h after transfection, luciferase activity was detected using a dual-luciferase reporter assay system (Promega, Madison, WI) and normalized to Renilla activity.

### Statistical analysis

All data from 3 independent experiments were expressed as mean ± SD and processed using SPSS 13.0 statistical software. The expression of miR-143 in CRC tissues and paired adjacent normal colonic tissues were compared by Wilcoxon's paired test. The difference among the groups in migration and invasion assay was estimated by Student's t-test or one-way ANOVA. A P-value of < 0.05 was considered to be statistically significant.

## Abbreviations

miR: microRNA; CRC: colorectal cancer; MACC1: metastasis-associated in colon cancer-1; 3'UTR: 3' untranslated regions.

## Competing interests

The authors declare that they have no competing interests.

## Authors' contributions

YZ and ZQW designed research and analyzed data. ZQW, MC, LP, XYW, QYM and FLM carried out molecular biology studies. YZ and BJ wrote the paper. All authors read and approved the final manuscript.
